# Anti-Alzheimer potential, metabolomic profiling and molecular docking of green synthesized silver nanoparticles of *Lampranthus coccineus* and *Malephora lutea* aqueous extracts

**DOI:** 10.1371/journal.pone.0223781

**Published:** 2019-11-06

**Authors:** Khayrya A. Youssif, Eman G. Haggag, Ali M. Elshamy, Mohamed A. Rabeh, Nagwan M. Gabr, Amany Seleem, M. Alaraby Salem, Ahmed S. Hussein, Markus Krischke, Martin J. Mueller, Usama Ramadan Abdelmohsen

**Affiliations:** 1 Department of Pharmacognosy, Faculty of Pharmacy, Modern University for Technology and Information, Cairo, Egypt; 2 Department of Pharmacognosy, Faculty of Pharmacy, Helwan University, Cairo, Egypt; 3 Department of Pharmacognosy, Faculty of Pharmacy, Cairo University, Cairo, Egypt; 4 Department of Pharmacology, National Research Centre, Cairo, Egypt; 5 Department of Pharmaceutical Chemistry, October University for Modern Sciences and Arts (MSA), Cairo, Egypt; 6 Julius-von-Sachs-Institute of Biosciences, Biocenter, Pharmaceutical Biology, University of Würzburg, Würzburg, Germany; 7 Department of Pharmacognosy, Faculty of Pharmacy, Minia University, Minia, Egypt; 8 Department of Pharmacognosy, Faculty of Pharmacy, Deraya University, Universities Zone, New Minia City, Minia, Egypt; VIT University, INDIA

## Abstract

The green synthesis of silver nanoparticles (SNPs) using plant extracts is an eco-friendly method. It is a single step and offers several advantages such as time reducing, cost-effective and environmental non-toxic. Silver nanoparticles are a type of Noble metal nanoparticles and it has tremendous applications in the field of diagnostics, therapeutics, antimicrobial activity, anticancer and neurodegenerative diseases. In the present work, the aqueous extracts of aerial parts of *Lampranthus coccineus* and *Malephora lutea* F. Aizoaceae were successfully used for the synthesis of silver nanoparticles. The formation of silver nanoparticles was early detected by a color change from pale yellow to reddish-brown color and was further confirmed by transmission electron microscope (TEM), UV–visible spectroscopy, Fourier transform infrared (FTIR) spectroscopy, dynamic light scattering (DLS), X-ray diffraction (XRD), and energy-dispersive X-ray diffraction (EDX). The TEM analysis of showed spherical nanoparticles with a mean size between 12.86 nm and 28.19 nm and the UV- visible spectroscopy showed λ_max_ of 417 nm, which confirms the presence of nanoparticles. The neuroprotective potential of SNPs was evaluated by assessing the antioxidant and cholinesterase inhibitory activity. Metabolomic profiling was performed on methanolic extracts of *L*. *coccineus* and *M*. *lutea* and resulted in the identification of 12 compounds, then docking was performed to investigate the possible interaction between the identified compounds and human acetylcholinesterase, butyrylcholinesterase, and glutathione transferase receptor, which are associated with the progress of Alzheimer’s disease. Overall our SNPs highlighted its promising potential in terms of anticholinesterase and antioxidant activity as plant-based anti-Alzheimer drug and against oxidative stress.

## 1. Introduction

Nanotechnology is a rapidly expanding multidisciplinary field, which deals with the understanding and regulating matter at a dimension of roughly 1 to 100 nanometers, and includes the understanding of the fundamental physics, chemistry, biology, and technology of nanometer-scale objects [1]. High surface areas of nanoparticles are responsible for their antimicrobial, magnetic, electronic and catalytic properties [2]. Nanoparticles of free metals have been extensively studied because of their unique physical properties, chemical reactivity and potential applications in catalysis, biological labeling, biosensing, drug delivery, antibacterial activity, antiviral activity, and detection of genetic disorders, gene therapy and DNA sequencing [3].

Nanoparticles have unique properties, which are quite different than those of larger particles. These new properties have been attributed to variations in specific characteristics such as size, shape, and distribution [4]. Silver (Ag), as a noble metal, with unique properties, it has potential applications in medicine [5]. There are various methods for SNPs preparation, such as the chemical precipitation, reverse micelle method, hydrothermal method, microwave, chemical vapor deposition, and biological methods. [6,7,8]. However, biological methods are preferred for being cost-effective and eco-friendly, as they don’t involve the use of toxic chemicals. Nanoparticles green synthesis is not time-consuming compared to other biological processes [9]. During the last 5 years, many efforts were put into developing newer and cheaper methods for synthesis of nanoparticles [10]. A very large number of microorganisms such as bacteria, fungi, yeasts, and plants has been discovered to have the ability to synthesize nanoparticles [10]. Accordingly, using vitamins, amino acids, microorganisms and plants extracts for the synthesis of nanoparticles is being greatly popularized nowadays and attracted high attention [11]. Pure enzymes with well-defined structure can be used for green synthesis of nanoparticles e.g. Ag nanoparticles were used to be combined by an enzyme induced growth process on strong substrates in nanoparticles synthesis. The enzyme was incorporated in polymer multilayer-assembled membranes through electrostatic interactions to develop the direct and “green” synthesis of bimetallic Fe/Pd particles in a membrane domain [12]. Nadagouda and Varma, reported the green combination of Ag and palladium nanospheres, nanowires, and nanorods by vitamin B_2_ as a reducing agent. Shivaji *et al*. developed stable SNPs in dark place for eight months by using cell-free culture supernatants of *psychrophilic bacteria*, *Pseudomonas antarctica*, *Pseudomonas proteolytica*, *Pseudomonas meridiana*, *Arthrobacter kerguelensis*, *Arthrobacter gangotriensis*, *Bacillus indicus* and *Bacillus cecembensis* [13]. Yeasts and fungi can also be used for the synthesis of SNPs, were silver nitrate was transformed into Ag oxide, forming well-dispersed SNPs, by the action of *Fusarium oxysporum*. The introduction of Ag particles to *Fusarium oxysporum* brought about the release of nitrate reductase ensuing development of extremely stable SNPs in solution [14].

Plant-based synthesis of NPs is certainly not a complicated procedure, where a metal salt is added with plant extract and the response is completed in minutes to a couple of hours at room temperature. This strategy has attracted more attention among the most recent advances particularly for silver (Ag) and gold (Au) NPs synthesis, which are more secure in comparison to other metallic NPs [15]. Synthesis of SNPs using different medicinal plants for pharmaceutical and biological applications have been reported [16,17,18,19,20,21,22]. Green synthesized nanoparticles have many therapeutic applications e. g. antimicrobial [23] (**S5 Table and S6 Table**), antioxidant [24], cytotoxic [25], and anti-inflammatory property [26]. Recently, nanotechnology played an important role in the development and improvement of techniques for the diagnosis and treatment of Al Alzheimer’s disease [27]. Several nanoparticles such as titanium dioxide, silica dioxide, silver and zinc oxide have been used for treatment of neurological disease [28], where oxidative nanoparticles can decrease the activities of reactive oxygen species (ROS) scavenging enzymes such as glutathione peroxidase (GSH-Px), superoxide dismutase (SOD) and catalase in the brain of rats and mice [28].

Moreover, intravenous administration of nanoparticles are promising delivery systems for the treatment of neurodegenerative diseases [29], e.g. In the case of Alzheimer’s disease, which is a form of dementia resulting in problems regarding memory, cognition, and behavior [30], biodegradable polymeric nanoparticles consisting of polyethylene glycol and/or poly(lactic-co-glycolic acid) and functionalized with specific antibodies [31,32] or oligopeptide drugs [33] have been used to eliminate and prevent the formation of amyloid fibrils, leading to this disease.

So this study was undertaken to investigate the possible anti-Alzheimer and antioxidant activity of the aqueous extracts of *L*. *coccineus* and *M*. *lutea*, along with investigating the phytochemical composition of the crude methanolic extracts of the two plants through UPLC-MS metabolomics profiling, followed by molecular docking in order to explore the chemical compounds that might contribute to the anti-Alzheimer and antioxidant activity.

## 2. Materials and methods

### 2.1. Plant material

Fresh aerial parts of *Lampranthus coccineus* and *Malephora lutea* were collected in September 2016 from Engineer Ahmed Helal farm, Sheikh Zayed, Cairo, Egypt, and authenticated by senior botanist Mrs. Therris Labib head specialist for plant taxonomy, El-Orman botanical garden, Giza, Egypt. The two plants were washed with tap water, and the surface washed with distilled water until no impurities remained. The clean aerial parts were shade dried for 20 days at room temperature to remove moisture. The dried aerial parts were pulverized in a clean electric blender to obtain a fine powder and stored in an airtight, amber glass bottle to avoid sunlight for further use.

### 2.2. Chemicals

All the reagents purchased were of analytical grade and used without any further purification. Silver nitrate (AgNO_3_) was purchased from Sigma-Aldrich, Germany with ≥ 99.5% purity and distilled water was used for the preparation of aqueous extracts for all experiments.

### 2.3. Human acetylcholinesterase ELISA kit

AChE was determined using NOVA human acetylcholinesterase (AChE) ELISA kit, Beijing, China, which uses Sandwich-ELISA as the method. The micro Elisa strip plate in this kit has been pre-coated with an antibody specific to AChE. Standards or samples are added to the appropriate Micro Elisa strip plate wells and combined to the specific antibody. Then a horseradish peroxide (HRP)-conjugated antibody specific for AChE is added to each well and incubated. Free components are washed away. The 3,3`,5,5`-Tetramethylbenzidine (TMB) substrate solution is added to each well. Only the wells that contain AChE and HRP conjugated AChE antibody will appear blue in color and then turn yellow after the addition of the stop solution. The optical density (OD) is measured spectrophotometrically using a double beam V-630 spectrophotometer, Jasco, Japan at a wavelength of 450 nm. The OD value is proportional to the concentration of AChE. And then comparing the OD of the samples to the standard curve.

### 2.4. Synthesis of silver nanoparticles using aqueous plant extract

Silver nanoparticles were synthesized by macerating 10 grams of the powdered plant in 100 ml distilled water, the mixture was kept in a water bath at 60°C for 30 min. Then the extract was filtered by Whatman no. 1 filter paper. For the biosynthesis of silver nanoparticles, the extract was added to 1 mM silver nitrate in the ratio 2:10 and kept in a water bath for 10 min at 60°C [34].

### 2.5. Metabolomic profiling of *Lampranthus coccineus* and *Malephora lutea* extracts

Metabolomic profiling was performed on methanolic extracts of *L*. *coccineus* and *M*. *lutea* according to Abdelmohsen et al. [35,36,37] on an Acquity Ultra Performance Liquid Chromatography system coupled to a Synapt G2 HDMS quadrupole time-of-flight hybrid mass spectrometer (Waters, Milford, USA). Chromatographic separation was carried out on a BEH C18 column (2.1 × 100 mm, 1.7 μm particle size; Waters, Milford, USA) with a guard column (2.1 x5 mm, 1.7 μm particle size) and a linear binary solvent gradient of 0%–100% eluent B over 6 min at a flow rate of 0.3 mL min−1, using 0.1% formic acid in water (v/v) as solvent A and acetonitrile as solvent B. The injection volume was 2 μL and the column temperature was 40°C. To convert the raw data into separate positive and negative ionization files, MSConvert software was used. The files were then imported to the data mining software MZmine 2.10 for peak picking, deconvolution, deisotoping, alignment and formula prediction 11. The database used for the identification of compounds was the Dictionary of Natural Products (DNP) 2015.

### 2.6. Anticholinesterase activity

**Fig 1:**
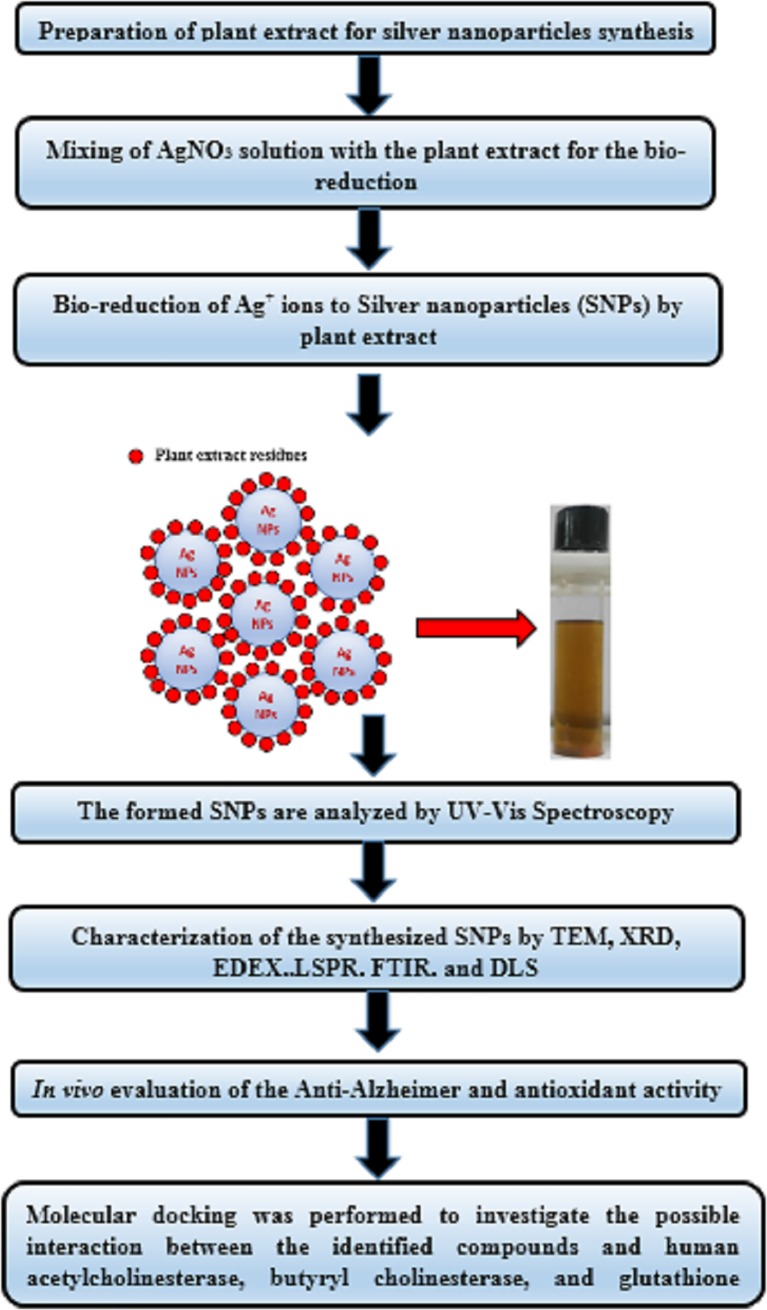
Experimental flow chart.

This study was conducted on adult male Albino rats of Sprague-Dawley of 130–150 g body weight in compliance with the guidelines for animal experiments set by the ethical committee of the National Research Centre and animals were treated in accordance with Canadian Council on Animal Care (CCAC). The unnecessary disturbance of animals was avoided. The animals were treated gently; squeezing, pressure and tough maneuver were avoided. The study was also approved by the Research Ethics Committee for Animal Experimentation, Department of Pharmacology and Toxicology, Faculty of Pharmacy, Helwan University, Egypt (project code 02A2019). They were kept under the same hygienic conditions and on a standard laboratory diet consisting of vitamin mixture (1%), mineral mixture (4%), corn oil (10%), sucrose (20%), cellulose (0.2%), pure casein (95%) and starch (54.3%). Animals were randomly classified into 8 groups each of 6 animals and treated according to the following scheme: **gp1:** received 1 ml saline and served as a normal healthy group, **gp2:** received 1 ml of 1mM AgNO_3_ and served as control group, **gp3:** received AlCl_3_ intraperitoneal (i.p.) 100mg/kg body weight (b.wt) once daily for 2 month and served as demented group, **gp4 & gp5:** received AlCl_3_ (i.p.) 100 mg/kg (b.wt)+ 20mg/kg *L*. *coccineus* aqueous extract and nanosilver aqueous respectively, **gp6 & gp7**: received AlCl_3_ (i.p.) 100 mg/kg (b.wt)+ 20mg/kg *M*. *lutea* aqueous extract and nanosilver aqueous respectively, and finally **gp8**: received AlCl_3_ (i.p.) 100 mg/kg (b.wt) + Rivastigmine drug (0.3 mg/kg) once daily for 2 months. After the treatment period the rats were anesthetized with Xylazine (10 mg/kg) and Ketamine (75 mg/kg) according to [38], and then the animals were sacrificed by decapitation and brains hippocampus were rapidly excised for each group, weighed and homogenized in ice-cold phosphate buffer to prepare 10% (w/v) homogenates and stored at 4°C for biochemical analysis. Data are presented as mean ± S.E from six animals in each group. Statistical significance was evaluated using the ANOVA test followed by post hoc Duncan's multiple range test. A probability value of less than 0.05 was considered statistically significant (P < 0.05), after synthesis and characterization of SNPS. The experimental work is summarized in a flow chart presented in (**Fig 1**)

### 2.7. Antioxidant activity

The antioxidant activity of all extracts was evaluated and compared with that of Rivastigmine after a daily dose of (0.3 mg/kg b.wt) for 2 months as a standard drug [39,40].

#### 2.7.1. Effect on brain malondialdehyde (MDA) in Alzheimer-induced rats

Thiobarbituric acid (TBA) reacts with malondialdehyde (MDA) in an acidic medium at a temperature of 95°C for 30 min. to form the Thiobarbituric acid reactive product. The absorbance of the resultant pink product can be measured at 534 nm [39,40].

Malondialdehyde (MDA) concentration in the sample:

Tissue = A _Sample_ / A _Standard_ X 10 / g Tissue used = nmol/g Tissue

#### 2.7.2. Effect on brain glutathione (GSH) in Alzheimer-induced rats

The method based on the reduction of 5, 5' dithiobis (2-nitrobenzoic acid) (DTNB) with glutathione (GSH) to produce a yellow compound. The reduced chromogen is directly proportional to GSH concentration and its absorbance can be measured at 405 nm. Prior to dissection, the tissue is perfused with a PBS (Phosphate buffered saline) solution, PH 7.4, containing 0.16 mg/ml heparin to remove any red blood cells and clots. Then the tissue is homogenized in 5–10 ml cold buffer (50 mM potassium phosphate, PH 7.5, 1 nM EDTA) per gram tissue using tissue homogenizer, and centrifugation at 4000 rpm for 15 minutes at 4°C. The supernatant is removed for assay and stored on ice.

Glutathione (GSH) concentration in sample:

In tissue = A _Sample_ X 2.22 / g Tissue used = μg/g tissue

### 2.8. Characterization of the synthesized SNPs by TEM

A drop of the silver nanoparticles solution was placed on a copper grid and coated with carbon support film. After drying, the shape and size of SNPs were analyzed using Transmission Electron Microscope (TEM), Jeol model JEM-1010, USA at The Regional Center for Mycology and Biotechnology, Al-Azhar University, Cairo, Egypt.

### 2.9. Characterization of the synthesized SNPs by UV-visible spectrometer

The formation of SNPs was monitored by measuring the UV–Vis spectrum of the reaction medium using a double beam V-630 spectrophotometer, Jasco, Japan, at the wavelength range from 200 to 600 nm at the College of Pharmacy, Ain Shams University, Cairo, Egypt.

### 2.10. Characterization of the synthesized SNPs using FTIR

FTIR-8400S, IR Prestige-21, IR Affinity-1, Shimadzu, Japan at College of Pharmacy, Cairo University, Cairo, Egypt, was used for characterization of the functional group attached to the surface of SNPs.

### 2.11. Determination of SNPs particle size distribution (Z-average mean) by Zeta sizer using DLS technique

The nanoparticles particle size distribution was studied using a Zeta-sizer Nano ZS (Malvern instruments) in a disposable cell at 25°C, and the results were analyzed using Zeta-sizer 7.01 software, United Kingdom.

### 2.12. X-ray diffraction (XRD) analysis

The crystalline nature of silver nanoparticles was checked by X-ray diffraction (XRD) analysis using an X-Ray diffractometer (Shimadzu Lab, XRD-6000, Japan). The information of translational symmetry-size and shape of the unit cell are obtained from peak positions of the diffraction pattern [41].

### 2.13. Energy dispersive X-ray spectroscopy (EDX) analysis

Energy-dispersive X-ray spectroscopy (EDX) of the synthesized SNPs was carried out using JED-2300T Energy Dispersive X-ray Spectrometer, USA at the Egyptian Atomic Energy Authority, Cairo, Egypt.

### 2.14. Molecular docking

Three crystal structures were selected to study the anti-Alzheimer activity of the ligands. The first crystal structure (PDB ID: 4BDS) is for Human butyryl cholinesterase. The 4BDS crystal has a co-crystallized ligand, tacrine (a cholinesterase inhibitor), that was utilized in defining the active site. The second crystal structure (PDB ID: 4M0E) is for human acetylcholinesterase with co-crystallized ligand (dihydrotanshinone I) that was used to define the active site. The third crystal structure (PDB ID: 4ZBD) is for glutathione transferase. The 4ZBD crystal’s binding site was defined by co-crystallized glutathione. Hence, three docking sites were used to study the binding patterns and affinities of the ligands. In all dockings, a grid box of dimensions 40 grid points and spacing 0.375 was centered on the given co-crystallized ligand. Four conformations were generated for each ligand using OpenBabel, and docking was performed via Autodock4 implementing 100 steps of the genetic algorithm while keeping all the default setting provided by Autodock Tools. Visualization was done using the Discovery studio program.

## 3. Results and discussion

### 3.1. TEM characterization of the synthesized SNPs of *L*. *coccineus* and *M*. *lutea* aqueous extracts

**Fig 2:**
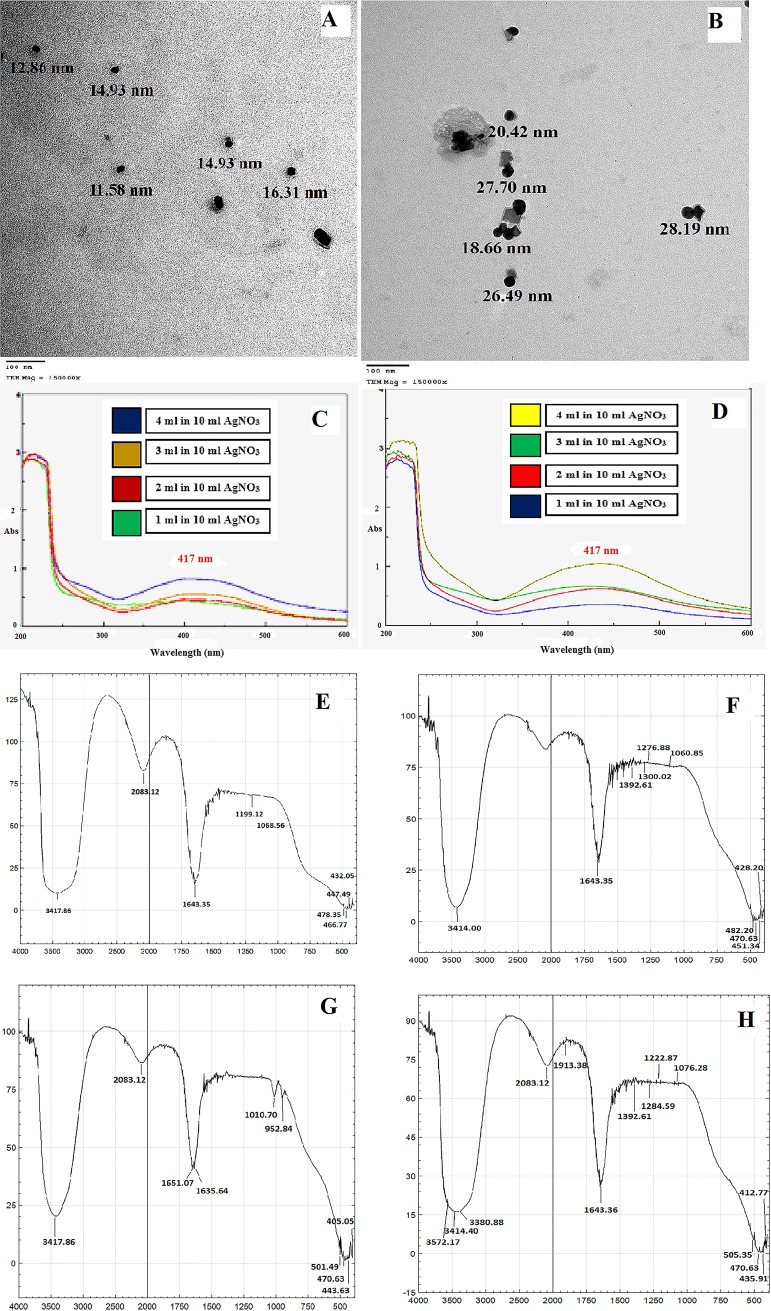
TEM photos for the shape and size of produced SNPs aqueous extracts of *Lampranthus coccineus* (A) and *Malephora lutea* (B) treated with AgNO3, UV-Vis. spectral analysis and color intensity of biosynthesized SNPs at various concentrations of *Lampranthus coccineus* (C) and *Malephora lutea* (D) aqueous extracts with AgNO_3_, FTIR spectra of *Lampranthus coccineus* aqueous extract before (E) and after (F) synthesis of nanoparticles, and *Malephora lutea* aqueous extract before (G) and after (H) synthesis of nanoparticles.

The TEM analysis of silver nanoparticles showed spherical nanoparticles with a mean size ranges between 12.86 nm to 28.19 nm. **(Fig 2A and 2B).**

### 3.2. UV-Visible characterization of the synthesized SNPs of *L*. *coccineus* and *M*. *lutea* aqueous

Ag echibit the highest efficiency of plasmon excitation among other metals e.g. Au and Cu. In addition, the optical excitation of plasmon resonance in nanosized Ag particles in the most efficient mechanism by which light interacts with matter, and it is known that silver is also the only material whose plasmon resonance can be tuned to any wavelength in the visible spectrum [42].

Synthesis of SNPs was first confirmed by changing their color to reddish-brown color due to surface plasmon resonance (SPR) phenomenon (**S1 Fig**) [43]. The formation of SNPs was monitored by measuring the UV–Vis spectrum **(Fig 2C and 2D)** of the reaction medium from 200 to 600 nm. UV-Vis spectroscopy can show that SPR peaks for green synthesized SNPs were between 370–435 nm [43]. In the current study, silver nanoparticles were synthesized at different concentrations of aqueous extracts of *L*. *coccineus* and *M*. *lutea* using 1 mM of silver nitrate. It has been observed that increasing the concentration of the aqueous extract resulted in increasing of the absorbance spectra with the small shifting of SPR peak to longer wavelength direction. At 4 ml concentration of the aqueous extract, the absorbance is maximum and the SPR band occurs at 417 nm [43]. The slight variations in the values of absorbance intensity signify that the changes are due to a change in the particle size [44, 45].

### 3.3. FTIR characterization of synthesized SNPs

FTIR has become an important tool in understanding the involvement of functional groups in the relation between metal particles and biomolecules which is used to search the chemical composition of the surface of the silver nanoparticles and identify the biomolecules for capping and efficient stabilization of the metal nanoparticles. There are many functional groups present which may have been responsible for the bio-reduction of Ag^+^ ions. FTIR spectrum (**Fig 2E, 2F, 2G and 2H)** show different major peak positions at 3572.17, 3421.72, 3414, 3390.86, 2083.12, 1913.39, 1643.35, 1635.64, 1392.61, 1284.59, 1222.87, 1246.02, 1222.87, 1076.28, 505.35, 470.63, 450.06, 435.91 and 428.20 cm^-1^. Those peaks indicate the presence of alcohol, phenolic and carboxylic acid compounds with strong hydrogen bonds and stretching of the N-H group. The FTIR peak in the range of 1635.64 cm^-1^ indicates, C = C bond stretching. The peak in the range 1392.61 cm^-1^ indicates the presence of tertiary amide, C-N stretching and N-H bending, and 1284.59–1222.87 cm^-1^ indicates C-O stretch in aromatic alcohols. Finally, the peaks in the range 1076.28 cm^-1^ indicates the presence of C-O and also aliphatic C-N bond stretching, while the peaks in the range 505.35–428.20 cm^-1^ demonstrate alkyl halides bond stretch. The observed peaks are mainly attributed to terpenoids, flavonoids, glycosides, phenols, and tannins with functional groups such as ketone, aldehyde, carboxylic acid, and others [46].

The presence of these groups increases the stability of the nanoparticles. These metabolites prevent aggregation and pairing of the nanoparticles. The similarity between the spectra with some marginal shifts in peak position clearly indicates the presence of the residual plant extract in the sample as a capping agent to the silver nanoparticles. Therefore, it may be inferred that these biomolecules are responsible for capping and efficient stabilization of synthesized nanoparticles [47].

### 3.4. Determination of the nanoparticles particle size distribution (Z-average mean) of *Lampranthus coccineus* and *Malephora lutea* aqueous and hexane extracts SNPs

**Fig 3:**
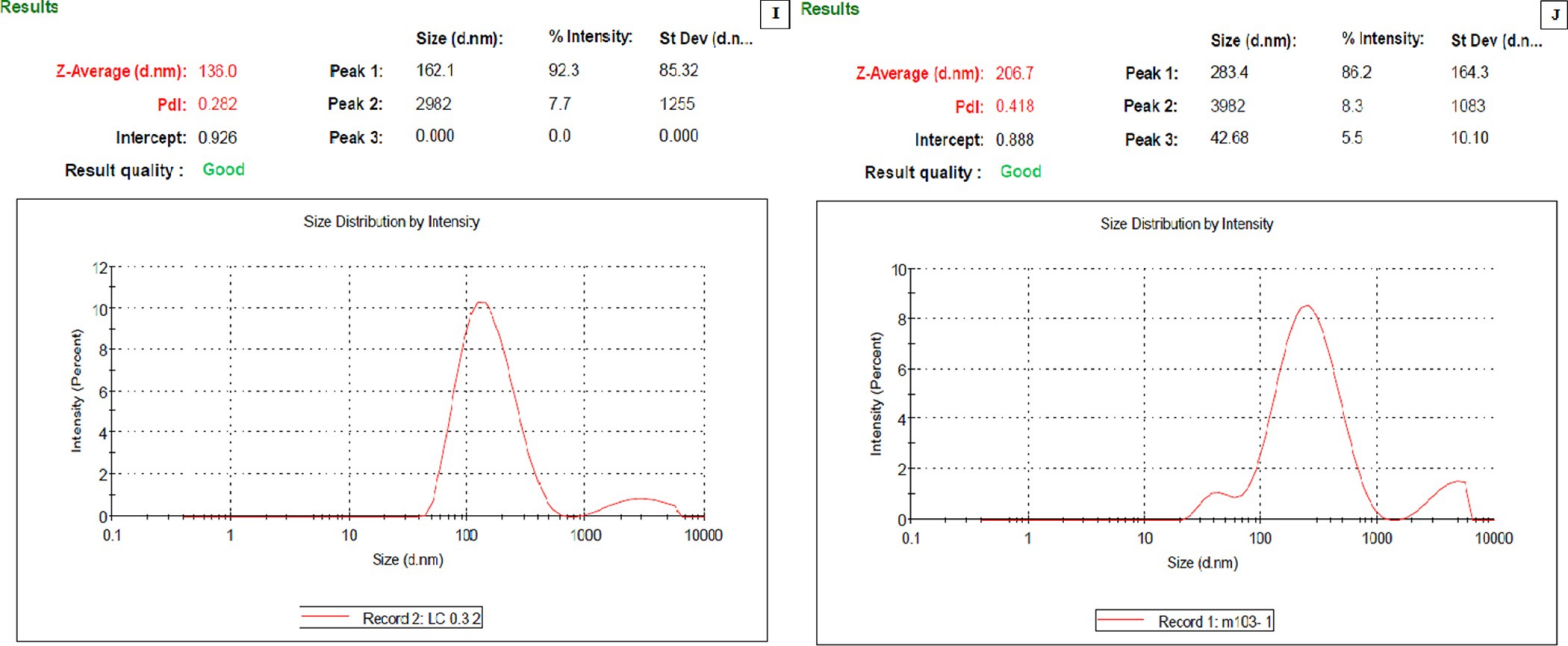
Dynamic light scattering analysis of synthesized SNPs (I) *L*. *coccineus* aqueous nano extract, (J) *M*. *lutea* aqueous nano extract.

Using DLS technique, the z-average mean (d.nm) in case of *L*. *coccineus* aqueous nano extract was 136 with a polydispersity index (PDI) 0.282 and in case of *M*. *lutea* aqueous nano extract with the z-average is 206.7 with a polydispersity index (PDI) 0.418. The morphology and dimensions of the biosynthesized SNPs were initially characterized using TEM image as shown in (**Fig 2A and 2B)** with an average size ranges from 12.86 nm to 28.19 nm. It should be noted that the mean particle size determined by TEM analysis was significantly smaller than that measured by DLS analysis (**Fig 2A and 2B** & **Fig 3I and 3J**). This contradiction could be possibly due to the adsorption of organic stabilizers from the extract on the surface of SNPs, the aggregation of some small particles and the adsorption of water on the stabilized SNPs [48,49,50].

### 3.5. X-ray diffraction (XRD) analysis

**Fig 4:**
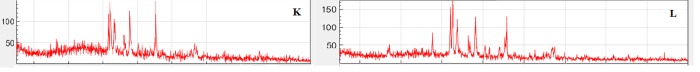
X-ray diffraction analysis (XRD) of synthesized SNPs (K) *L*. *coccineus* aqueous nano extract, (L) *M*. *lutea* aqueous nano extract.

The dry powders of the green synthesized SNPs were subjected to XRD analysis. The diffracted intensities were recorded from 4° to 90° at 2 theta angles. The X-ray diffraction (XRD)spectra are used to confirm the crystalline nature of the synthesized SNPs by using *L*. *coccineus* and *M*. *lutea* aqueous extracts and the patterns are exhibited in (**Fig 4K and 4L**). The spectra of XRD clearly indicate that the synthesized silver nanoparticles using the above-mentioned extracts are crystalline in nature.

The Bragg reflections of silver nanoparticles are observed at (2Ɵ value) that corresponds to 31.9°, 32.5°, 36.4°, 38.06°, 54.6° [51]. No peaks of the XRD spectra of Ag2O and other substances appear in (**Fig 4K and 4L**), and it can be stated that the obtained SNPs had a high purity, and the observed peak broadening and noise were most probably due to the effect of nanoparticles and the presence of various crystalline biological macromolecules in the aqueous extracts of *L*. *coccineus* and *M*. *lutea*. All the obtained results confirm that silver ions had been reduced to Ag^0^ by the aqueous extracts of *L*. *coccineus* and *M*. *lutea* under the reaction conditions [52].

### 3.6. Energy dispersive X-ray spectroscopy (EDX) analysis

**Fig 5:**
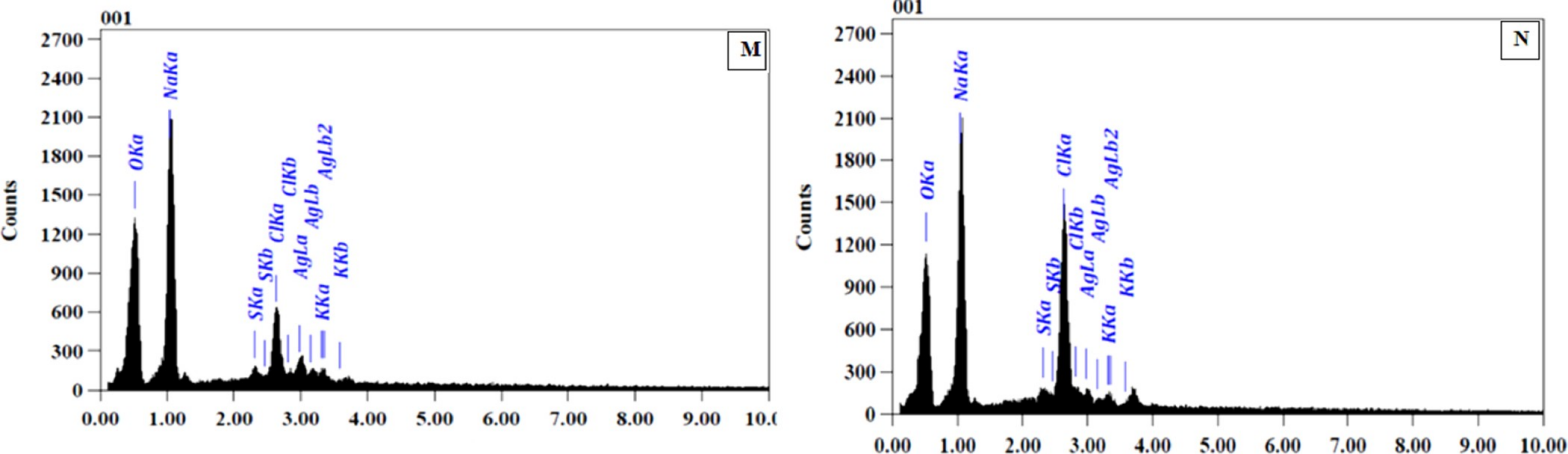
Energy-dispersive X-ray analysis (EDX) of synthesized SNPs (M) *L*. *coccineus* aqueous nano extract, (N) *M*. *lutea* aqueous nano extract.

The freeze-dried silver nanoparticles of *L*. *coccineus* and *M*. *lutea* aqueous extracts were mounted on specimen stubs with double-sided taps, coated with gold in a sputter coater, and examined under a JED-2300T Energy Dispersive X-ray Spectrometer at 30 kV. EDX spectrophotometer analysis of both the two samples established the existence of element Ag signal of SNPs and the homogenous distribution of it. It revealed a strong signal of Ag in the Ag region and is in **(Fig 5M and 5N)**. Metal silver nanocrystals generally showed a typical optical absorption peak approximately at 2.983 keV. There were other peaks for O, Na, S, Cl, and K suggesting that they are mixed precipitates present in the plant extract [52, 53].

### 3.7. Metabolomic profiling of the crude methanolic extracts of *L*. *coccineus* and *M*. *lutea*

**Fig 6:**
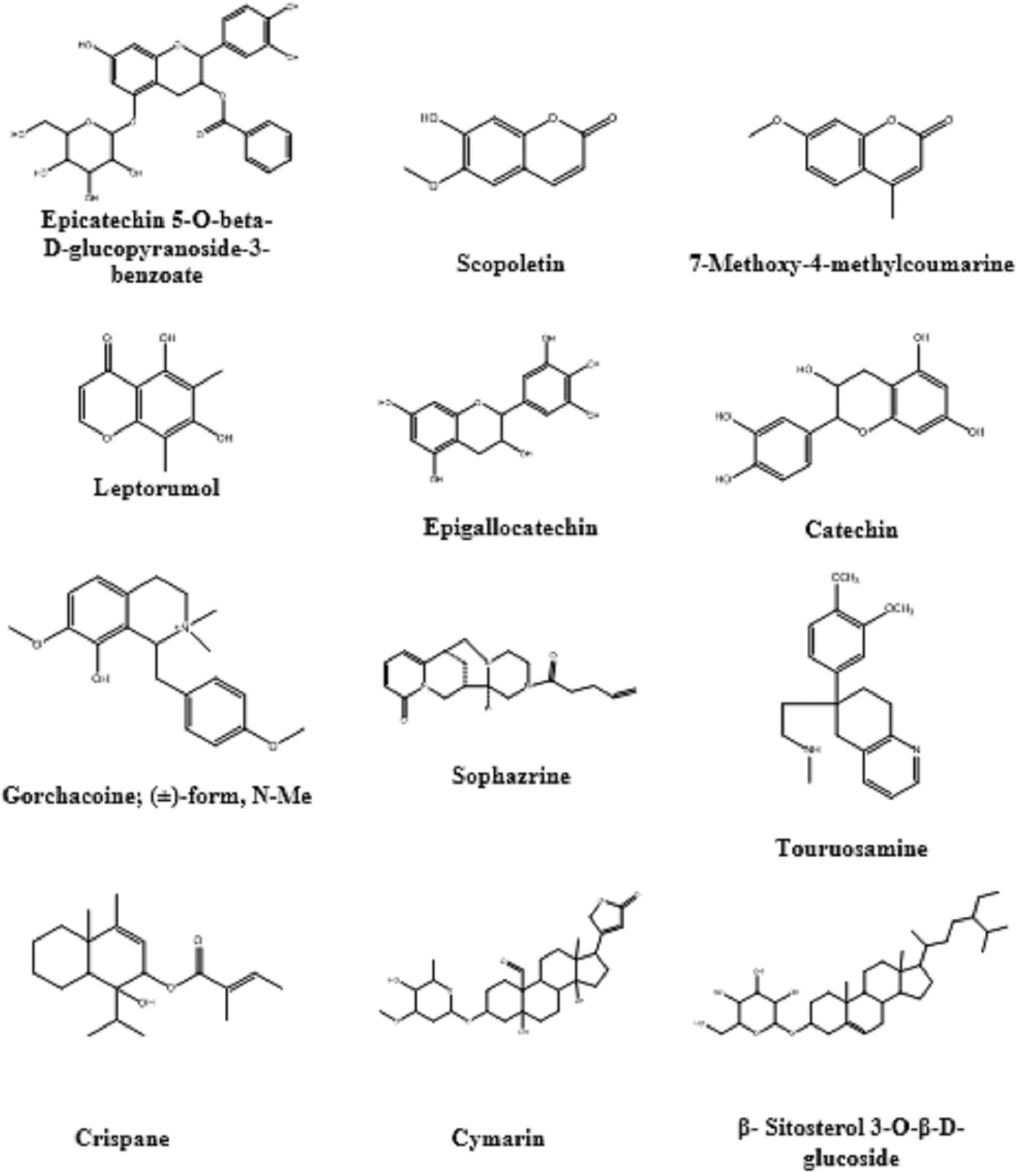
Compounds identified and dereplicated from the methanolic extract of *Lampranthus coccineus* and *Malephora lutea*.

Dereplication of the secondary metabolites from the crude methanolic extract of *L*. *coccineus* and *M*. *lutea* resulted in the identification of different classes of compounds including alkaloids, flavonoids, and steroidal compounds (**S1 Table**) and (**Fig 6**).

### 3.8. Anticholinesterase activity

The anti-Alzheimer activity of *L*. *coccineus* and *M*. *lutea* aqueous and aqueous nano extracts were evaluated and compared with that of Rivastigmine as standard using [54].

The *L*. *coccineus* aqueous and aqueous nano extracts showed the highest antiacetylcholinesterase activity (1.23, 0.82 ng/ml) respectively, followed by aqueous and nano extracts of *M*. *lutea* (1.95, 1.36 ng/ml) in comparison to the standard drug Rivastigmine (0.79 ng/ml) as shown in **(Table 1)** and **(S2 Fig)**.

**Table 1:** Effect of *Lampranthus coccineus*, *Malephora lutea* extracts and Rivastigmine drug on brain acetylcholinestrase (AChE) in Alzheimer's disease induced rats.

Group	Acetylcholinestrase ng/ml (mean ± S.E)
Normal (saline)	0.73 ± 0.01
AgNO_3_	0.74 ± 0.01
AlCl_3_ (100 g / kg)	4.82 ± 0.01*
AlCl_3_ + *Lampranthus coccineus* aqueous extract.	1.23 ± 0.01*^a^
AlCl_3_ + *Lampranthus coccineus* nanosilver aqueous extract.	0.82 ± 0.03^a^
AlCl_3_ + *Malephora lutea* aqueous extract.	1.95 ± 0.01*^a^
AlCl_3_ + *Malephora lutea* nanosilver aqueous extract.	1.36 ± 0.01*^a^
Rivastigmine (0.3 mg/kg)	0.79± 0.01^a^

S.E: Standard error; groups consists of rats (6 rats each)

* Statistically significantly different from the control group at p <0.05.

a Statistically significantly different from the aluminum group at p <0.05

### 3.9. Antioxidant activity

#### 3.9.1. Effect of *L*. *coccineus*, *M*. *lutea* extracts, and Rivastigmine drug on brain malondialdehyde (MDA) in Alzheimer's disease-induced rats

The antioxidant activity of *L*. *coccineus* and *M*. *lutea* aqueous and aqueous nano extracts were evaluated by measuring the amount of malondialdehyde (MDA) and compared with that of Rivastigmine as standard using [39,40]. The aqueous nano extract of *L*. *coccineus* and *M*. *lutea* showed the highest antioxidant activity (36.4, 43.6 nmol/g tissue) respectively, followed by aqueous extract of *L*. *coccineus* (45.7 nmol/g tissue) and *M*. *lutea* (54.8 nmol/g tissue) in comparison to Rivastigmine (34.9 nmol/g tissue) (**S2 Table**) and (**S3 Fig**).

#### 3.9.2. Effect of *L*. *coccineus*, *M*. *lutea* extracts, and Rivastigmine drug on brain glutathione (GSH) in Alzheimer's disease induced rats

The antioxidant activity of *L*. *coccineus* and *M*. *lutea* aqueous and aqueous nano were evaluated by measuring the amount of reduced glutathione (GSH) and compared with that of Rivastigmine as standard [54, 55].

The aqueous and aqueous nano extract of *L*. *coccineus* and *M*. *lutea* showed the highest antioxidant activity (3.36, 3.16 μg/g tissue) respectively, followed by aqueous extracts of *L*. *coccineus* (2.91 μg/g tissue) and *M*. *lutea* (2.65 μg/g tissue) in comparison to Rivastigmine (3.68 μg/g tissue) (**S3 Table**) and (**S4 Fig**).

### 3.10. Molecular docking

**Fig 7:**
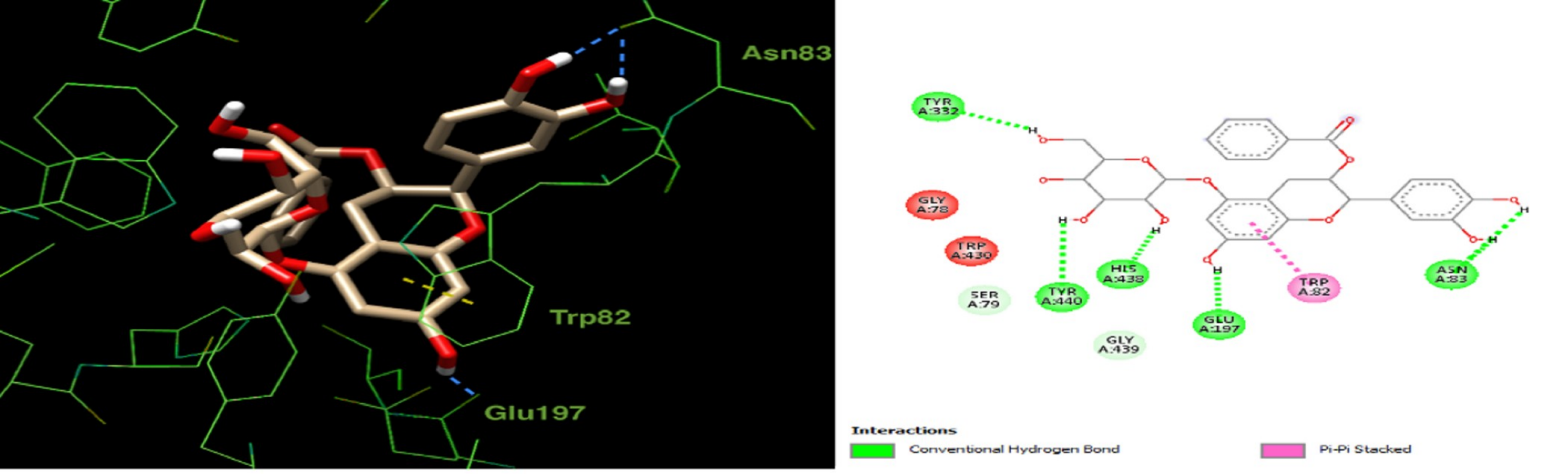
2D and 3D interaction plot for Epicatechin5-O-beta-D-glucopyranoside-3-benzoate. In the active site of butyrylcholinesterase (4BDS). Blue and yellow dashed lines refer to hydrogen bonds and π-π stacking, respectively.

**Fig 8:**
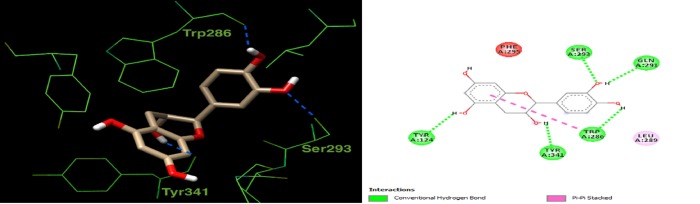
2D and 3D interaction plot for catechin in the active site of acetylcholinesterase (4M0E). Hydrogen bonds are shown as blue dashed lines.

**Fig 9:**
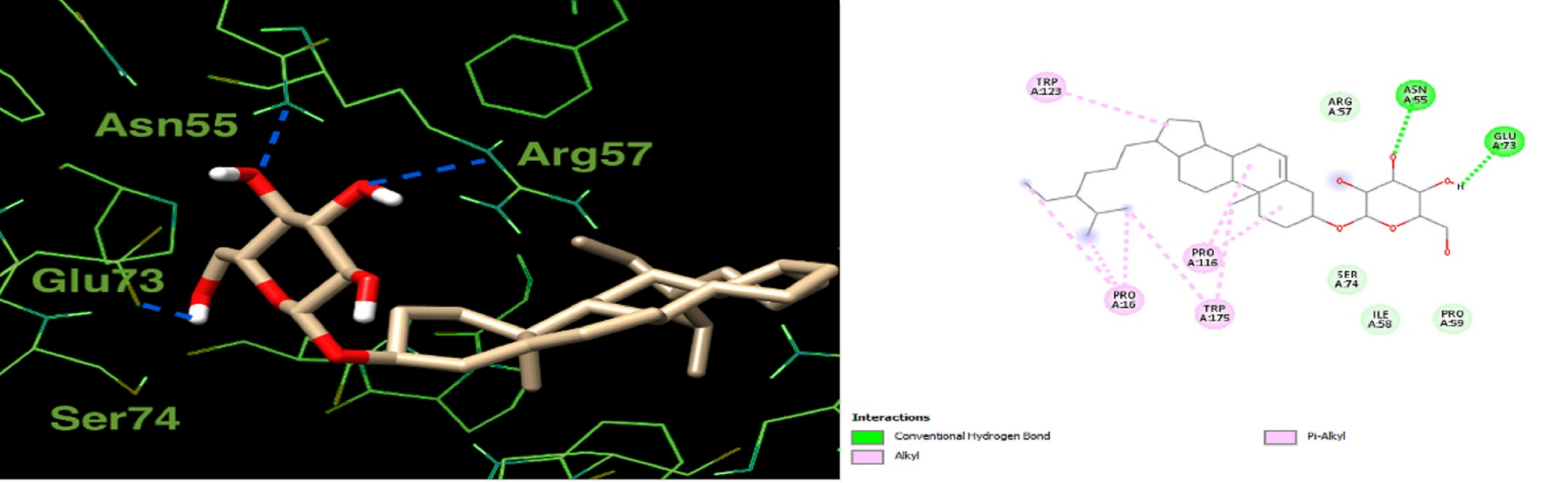
2D and 3D interaction plot for β-Sitosterol 3-O-β-D-glucoside in the active site of glutathione transferase (4ZBD). Hydrogen bonds are shown as blue dashed lines.

Docking all components of the *L*. *coccineus* and *M*. *lutea* methanolic extract into macromolecular targets involved in Alzheimer’s pathophysicology explain the observed anti-Alzheimer’s activity. Docking scores for the top-scoring compounds are given in (**S4 Table**). Further, 3D depictions showing the interactions of one top-scoring molecule with the surrounding amino acids in the active site of each protein target is given in (**Figs 7, 8 and 9**).

For butyrylcholinesterase, the co-crystallized ligand in the crystal structure (4BDS) mainly made π-π interactions with a neighboring tryptophan (Trp82). While several compounds in the extract have high scores, see (**S4 Table**), Epicatechin5-O-beta-D-glucopyranoside-3-benzoate shows the most similar docking poses to the co-crystallized ligand. As shown in (**Fig 7**), it is predicted to interact similarly with the same Trp82 in the active site. In addition, it interacts *via* H-bonds with Asn83 and Glu197.

For acetylcholinesterase, the co-crystallized ligand in the crystal structure (4M0E) made π-π interactions with a neighboring tryptophan (Trp286) and H-bonds with Phe295 and Tyr124. Catechin is also predicted to interact with Trp286, but *via* H-bonds, see (**Fig 8**). In addition, it forms H-bonds with Ser293 and Tyr341.

Finally, for glutathione transferase, the co-crystallized ligand in the crystal structure (4ZBD) made H-bonds with Arg57, Trp157, and Arg138. β- Sitosterol 3-O-β-D-glucoside forms an equivalent network of H-bonds with Arg57, Asn55, Glu73, and Ser74, as illustrated in (**Fig 9**).

In conclusion, the high scores and very similar interaction patterns of several ligands in the *L*. *coccineus* and *M*. *lutea* methanolic extract with known inhibitors of 3 enzymes involved in the pathophysiology of Alzheimer’s disease provide a molecular explanation for the anti-Alzheimer’s activity of the extract.

## 4. Conclusion

The formation of the synthesized SNPs was confirmed by observing the color change from pale yellow to reddish-brown color, and it has been also confirmed by using different techniques e.g. TEM, UV-Visiblespectroscopy, FTIR, DLS, XRD, and EDX. The results obtained from the present study confirm the formation of spherical nanoparticles with a mean size range between 12.86 nm to 28.19 nm estimated using transmission electron microscope (TEM), and The XRD spectra confirmed that silver ions had been reduced to Ag^0^ by the aqueous extracts of *L*. *coccineus* and *M*. *lutea* and it is crystalline in nature. The phytochemical constituents of methanolic extract of *L*. *coccineus* and *M*. *lutea* were characterized using UPLC-MS. A total of 12 compounds were identified, and their neuroprotective activity and antioxidant effect against AlCl_3_ induced Alzheimer’s disease in rats were evaluated. The nanosilver aqueous extracts of *L*. *coccineus* and *M*. *lutea* showed the highest antiacetylcholinesterase and antioxidant activity followed by the aqueous extracts of *L*. *coccineus* and *M*. *lutea*, which confirms that nanoparticles were able to cross the blood-brain barrier in the *in vivo* experiment and increase the level of acetylcholinesterase and decrease the level of oxidative stress. A molecular modeling study was also conducted to provide an insight regarding the molecular target proteins acetylcholinesterase, butyrylcholinesterase and glutathione transferase, that could be involved in the mechanism of action of the studied extracts. The docking results suggest that epicatechin5-O-beta-D-glucopyranoside-3-benzoate, catechin, and β-Sitosterol 3-O-β-D-glucoside serve as inhibitors for butyrylcholinesterase, acetylcholinesterase, and glutathione transferase, respectively.

In conclusion, *L*. *coccineus* and *M*. *lutea* aqueous nano extracts counteract oxidative stress and can be useful in treating Alzheimer’s disease.

## Supporting information

S1 Fig A, B. Early sign for the formation of SNPs in *Lampranthus coccineus* and *Malephora lutea* aqueous extracts, respectively observed as color change from pale yellow to reddish-brown color after 24 hours incubation with 1 mM AgNO_3_ solution.
(TIF)

S2 Fig Effect of *Lampranthus coccineus*, *Malephora lutea* aqueous extracts and Rivastigmine drug on brain acetylcholinestrase (AChE) in Alzheimer's disease induced rats. (TIF)

S3 Fig Effect of *Lampranthus coccineus*, *Malephora lutea* extracts plant and Rivastigmine drug on brain malondialdehyde (MDA) in Alzheimer's disease induced rats. (TIF)

S4 Fig Effect *Lampranthus coccineus*, *Malephora lutea* extracts and Rivastigmine drug on brain glutathione (GSH) in Alzheimer's disease induced rats. (TIF)

S1 Table Dereplication of the metabolomics of methanolic extract of *Lampranthus coccineus* and *Malephora lutea*. (DOCX)

S2 Table Effect of *Lampranthus coccineus*, *Malephora lutea* extracts plant and Rivastigmine drug on brain malondialdehyde (MDA) in Alzheimer's induced disease. (DOCX)

S3 Table Effect *Lampranthus coccineus*, *Malephora lutea* extracts and Rivastigmine drug on brain glutathione (GSH) in Alzheimer's disease induced rats. (DOCX)

S4 Table The scores of re-docking the co-crystallized ligands and the top-scoring compounds of the *Lampranthus coccineus* and *Malephora lutea* methanolic extract in the active sites of butyrylcholinesterase (4BDS), acetylcholinesterase (4M0E) and glutathione transferase (4ZBD). (DOCX)

S5 Table Antimicrobial activity as indicated by growth-inhibition zone in (mm) of *L*. *coccineus* and *M*. *lutea* aqueous and aqueous nano extracts against different strains of bacteria. (DOCX)

S6 Table Antimicrobial activity as MICS (μg/ml) of tested samples against tested microorganisms was performed for the most active samples. (DOCX)
