# Supplementary figures and images for: Anti-Alzheimer potential, metabolomic profiling and molecular docking of green synthesized silver nanoparticles of Lampranthus coccineus and Malephora lutea aqueous extracts
Source: PLoS One. 2019 Nov 6;14(11):e0223781. doi: 10.1371/journal.pone.0223781 (PMC6834257; doi:10.1371/journal.pone.0223781)

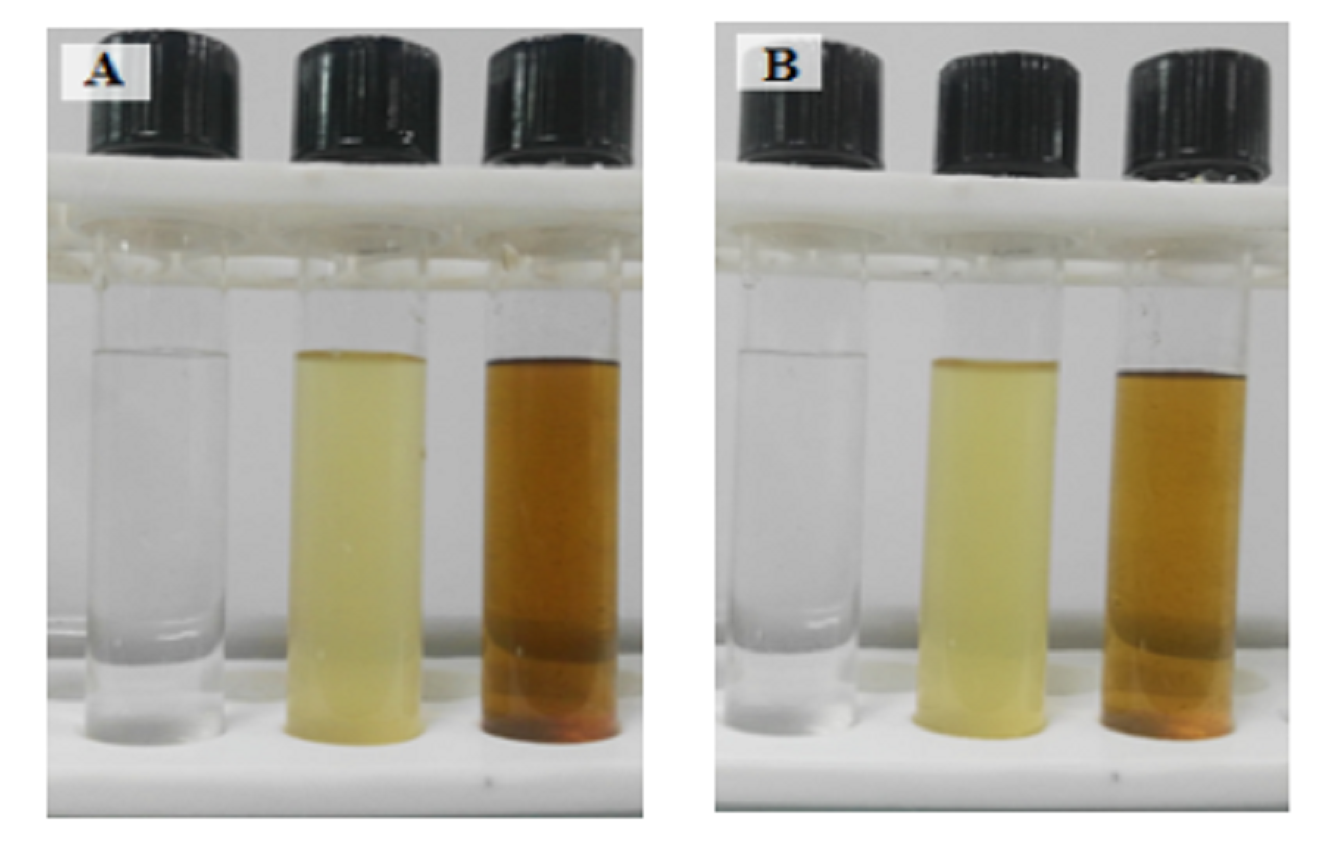

Supplement: S1 Fig — A, B. Early sign for the formation of SNPs in Lampranthus coccineus and Malephora lutea aqueous extracts, respectively observed as color change from pale yellow to reddish-brown color after 24 hours incubation with 1 mM AgNO3 solution. (TIF) [file pone.0223781.s001.tif]

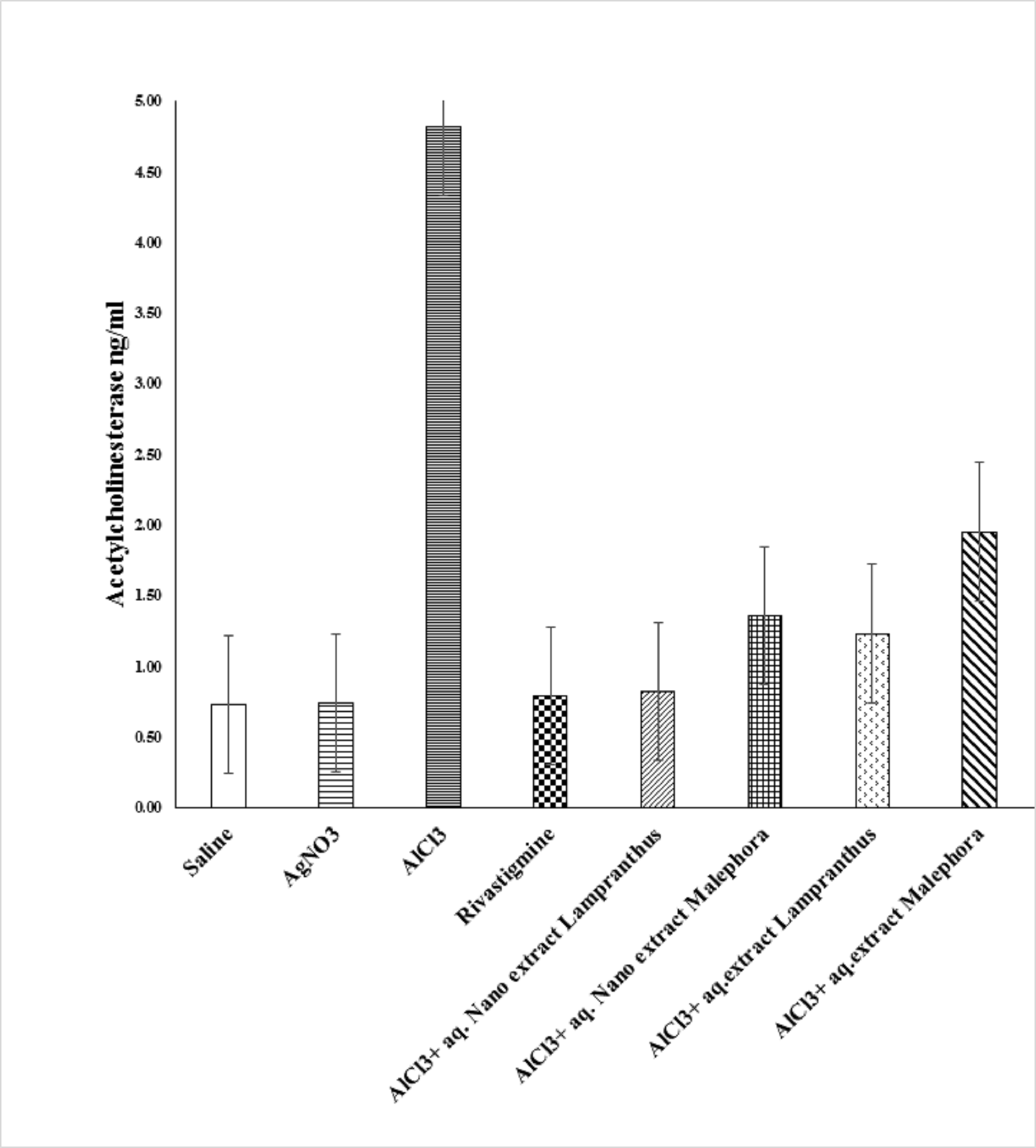

Supplement: S2 Fig — (TIF) [file pone.0223781.s002.tif]

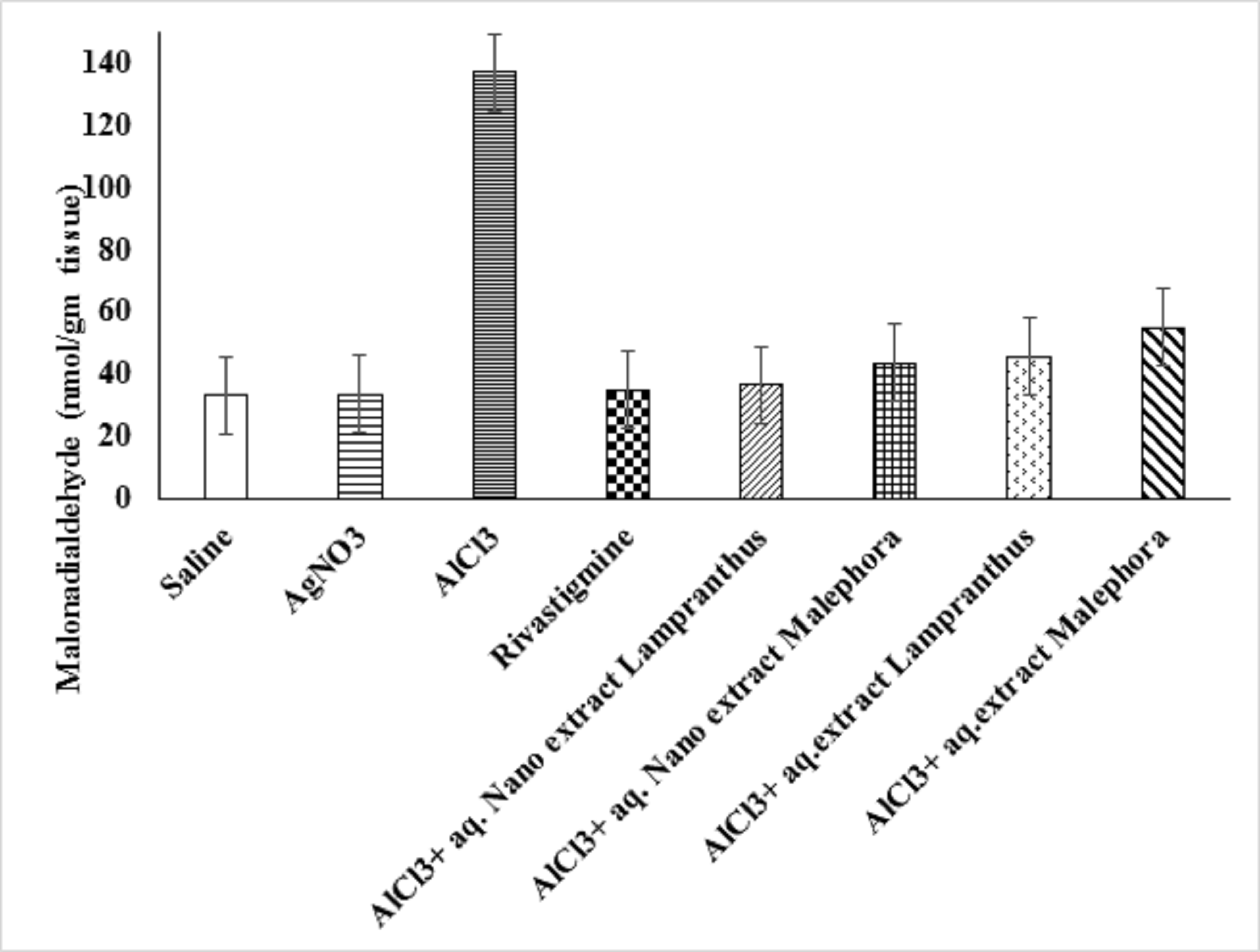

Supplement: S3 Fig — (TIF) [file pone.0223781.s003.tif]

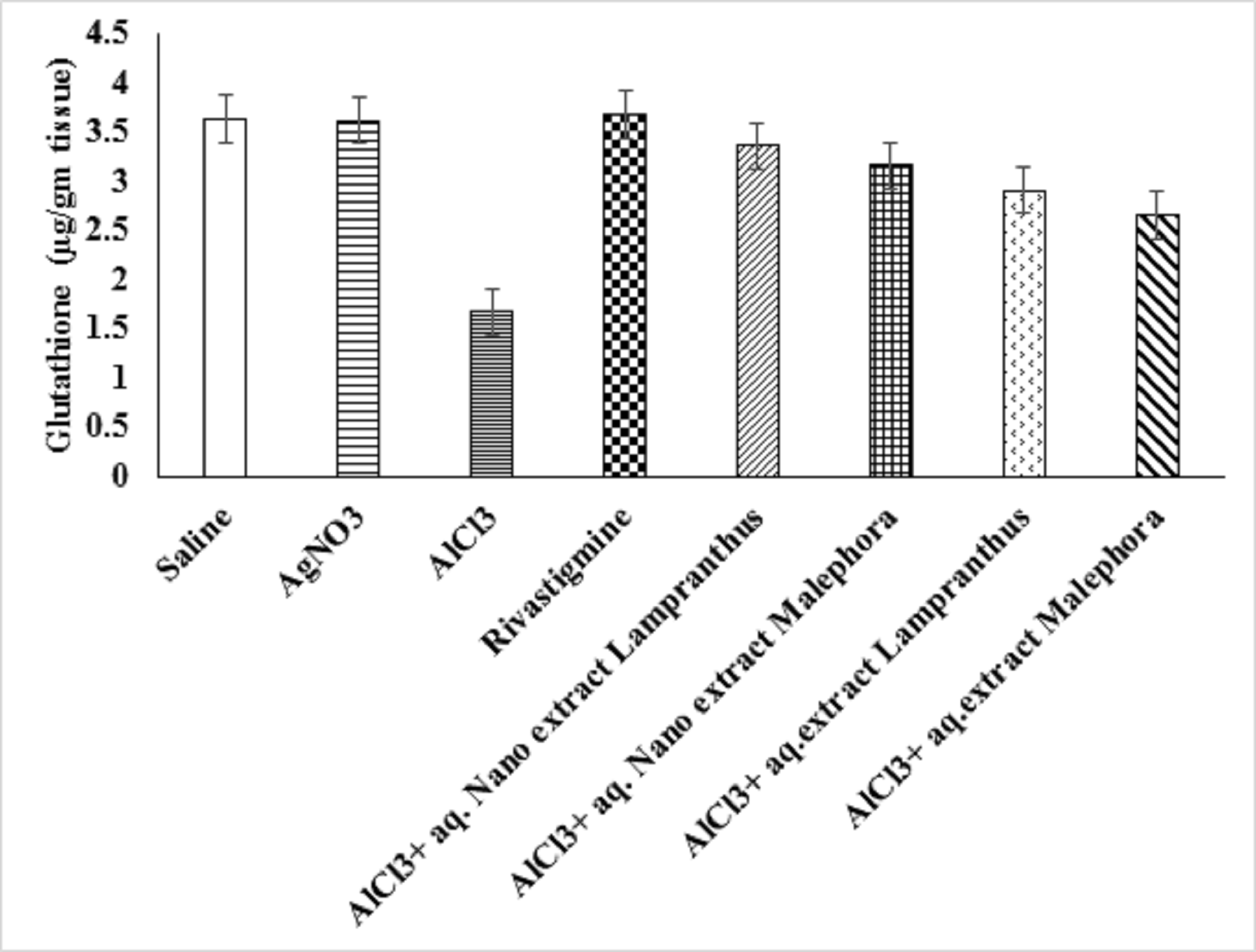

Supplement: S4 Fig — (TIF) [file pone.0223781.s004.tif]
