# Supplementary material for: Anti-Alzheimer potential, metabolomic profiling and molecular docking of green synthesized silver nanoparticles of Lampranthus coccineus and Malephora lutea aqueous extracts
Source: PLoS One. 2019 Nov 6;14(11):e0223781. doi: 10.1371/journal.pone.0223781 (PMC6834257; doi:10.1371/journal.pone.0223781)
Supplement: S1 Table — (DOCX) [file pone.0223781.s005.docx]

| **M/Z** | **Rt. (min.)** | **M.wt.** | **Name** | **Molecular formula** | **References** |
| --- | --- | --- | --- | --- | --- |
| 557.225 | 1 | 556.2175 | Epicatechin 5-O-beta-D-glucopyranoside-3-benzoate | [C_28_H_28_O_12_](https://pubchem.ncbi.nlm.nih.gov/search/#query=C28H28O12) | [1] |
| 191.019 | 1.7 | 192.0264 | Scopoletin | [C_10_H_8_O_4_](https://pubchem.ncbi.nlm.nih.gov/search/#query=C10H8O4) | [2] |
| 189.040 | 1.8 | 190.0471 | 7-Methoxy-4-methylcoumarine | C_11_H_10_O_3_ | [2] |
| 205.071 | 2.2 | 206.0783 | Leptorumol | [C_11_H_10_O_4_](https://pubchem.ncbi.nlm.nih.gov/search/#query=C11H10O4) | [3] |
| 305.106 | 2.3 | 306.1129 | Epigallocatechin | C_15_H_14_O_7_ | [4] |
| 289.110 | 2.8 | 290.1173 | Catechin | [C_15_H_14_O_6_](https://pubchem.ncbi.nlm.nih.gov/search/#query=C15H14O6) | [5] |
| 327.183 | 4.7 | 328.1904 | Gorchacoine; (±)-form, N-Me | C_20_H_26_NO_3_ | [6] |
| 326.187 | 4.8 | 327.1941 | Sophazrine | C_19_H_25_N_3_O_2_ | [7] |
| 325.184 | 4.8 | 326.1912 | Touruosamine | C_20_H_26_N_2_O_2_ | [8] |
| 305.215 | 5.2 | 306.2222 | Crispane | C_20_H_32_O_3_ | [9] |
| 547.367 | 5.9 | 548.3735 | Cymarin | C_30_H_44_O_9_ | [10] |
| 561.382 | 6 | 562.3889 | β-Sitosterol 3-O-β-D-glucoside | [C_35_H_60_O_6_](https://pubchem.ncbi.nlm.nih.gov/search/#query=C35H60O6) | [11] |
