# Supplementary material for: Anti-Alzheimer potential, metabolomic profiling and molecular docking of green synthesized silver nanoparticles of Lampranthus coccineus and Malephora lutea aqueous extracts
Source: PLoS One. 2019 Nov 6;14(11):e0223781. doi: 10.1371/journal.pone.0223781 (PMC6834257; doi:10.1371/journal.pone.0223781)
Supplement: S2 Table — (DOCX) [file pone.0223781.s006.docx]

| **Group** | **MDA (nmol/g tissue)**  **mean ± S. E** |
| --- | --- |
| Normal (saline) | 33.2 ± 1.2 |
| AgNO_3_ | 33.6 ± 1.4 |
| AlCl_3_ (100 mg / kg) | 137.2 ± 5.9^*^ |
| AlCl_3_ + *Lampranthus coccineus* aqueous extract. | 45.7 ± 2.2^*a^ |
| AlCl_3_ + *Lampranthus coccineus* nanosilver aqueous extract. | 36.4 ± 1.6^*a^ |
| AlCl_3_ + *Malephora lutea* aqueous extract. | 54.8 ± 2.4^*a^ |
| AlCl_3_ + *Malephora lutea* nanosilver aqueous extract. | 43.6 ± 1.9^*a^ |
| Rivastigmine (0.3 mg/kg) | 34.9 ± 1.3^a^ |

S.E: Standard error; groups consists of rats (6 rats each)

* Statistically significant different from control group at p <0.05.

a Statistically significant different from aluminum group at p <0.05
