# Supplementary material for: Anti-Alzheimer potential, metabolomic profiling and molecular docking of green synthesized silver nanoparticles of Lampranthus coccineus and Malephora lutea aqueous extracts
Source: PLoS One. 2019 Nov 6;14(11):e0223781. doi: 10.1371/journal.pone.0223781 (PMC6834257; doi:10.1371/journal.pone.0223781)
Supplement: S3 Table — (DOCX) [file pone.0223781.s007.docx]

| **Groups** | **GSH (µg/g tissue)  mean ± S. E** |
| --- | --- |
| Normal (saline) | 3.63 ± 0.03 |
| AgNO_3_ | 3.62 ± 0.01 |
| AlCl_3_ (100 mg / kg) | 1.67 ± 0.02^a^ |
| AlCl_3_ + *Lampranthus coccineus* aqueous extract. | 2.91 ± 0.01^*a^ |
| AlCl_3_ + *Lampranthus coccineus* nanosilver aqueous extract. | 3.36 ± 0.03^*a^ |
| AlCl_3_ + *Malephora lutea* aqueous extract. | 2.65 ± 0.01^*a^ |
| AlCl_3_ + *Malephora lutea* nanosilver aqueous extract. | 3.16 ± 0.03^*a^ |
| Rivastigmine (0.3 mg/kg) | 3.68 ± 0.04 ^a^ |

S.E: Standard error; groups consists of rats (6 rats each)

* Statistically significant different from control group at p <0.05.

a Statistically significant different from aluminum group at p <0.05
