# Supplementary material for: Anti-Alzheimer potential, metabolomic profiling and molecular docking of green synthesized silver nanoparticles of Lampranthus coccineus and Malephora lutea aqueous extracts
Source: PLoS One. 2019 Nov 6;14(11):e0223781. doi: 10.1371/journal.pone.0223781 (PMC6834257; doi:10.1371/journal.pone.0223781)
Supplement: S4 Table — (DOCX) [file pone.0223781.s008.docx]

|  | **Ligand** | **4BDS** | **4M0E** | **4ZBD** |
| --- | --- | --- | --- | --- |
| Cocrystallized  Compounds from the *Lampranthus coccineus* and *Malephora lutea* methanolic extract | Tacrine | -7.32 |  |  |
|  | Dihydrotanshinone I |  | -9.26 |  |
|  | Glutathione |  |  | -6.26 |
|  | 7-Methoxy-4-methylcoumarine | -6.05 | -6.61 | -6.03 |
|  | Catechin | -8.16 | -7.59 | -7.03 |
|  | Cymarin | -8.15 | -10.21 | -8.31 |
|  | Epicatechin5-O-beta-D-glucopyranoside-3-benzoate | -10.9 | -9.68 | -8.43 |
|  | Epigallocatechin | -7.6 | -7.91 | -6.95 |
|  | Scopoletin | -5.78 | -6.29 | -6.1 |
|  | β-Sitosterol 3-O-β-D-glucoside | -9.29 | -10.97 | -9.28 |
|  | Sophazrine | -9.17 | -9.22 | -5.93 |
