# Supplementary material for: Anti-Alzheimer potential, metabolomic profiling and molecular docking of green synthesized silver nanoparticles of Lampranthus coccineus and Malephora lutea aqueous extracts
Source: PLoS One. 2019 Nov 6;14(11):e0223781. doi: 10.1371/journal.pone.0223781 (PMC6834257; doi:10.1371/journal.pone.0223781)
Supplement: S5 Table — (DOCX) [file pone.0223781.s009.docx]

| **Samples**  **Tested Microorganisms** | **AgNO_3_** | ***L. coccineus* aq. extract** | ***L. coccineus* aq. nano extract** | ***M. lutea* aq. extract** | ***M. lutea* aq. nano extract** | **Standard antimicrobial agents** |
| --- | --- | --- | --- | --- | --- | --- |
| **Gram positive bacteria:** |  |  |  |  |  | Gentamycin |
| *Staphylococcus aureus* | NA | 9 | 15 | 14 | 17 | 24 |
| *Bacillus sphaericus* | NA | 13 | 17 | 13 | 17 | 26 |
| **Gram negative bacteria:** |  |  |  |  |  | Gentamycin |
| *Enterobacter aerogenes* | NA | 12 | 16 | NA | 18 | 26 |
| *Pseudomonas aeruginosa* | NA | 10 | 13 | NA | 13 | 30 |
| **Fungi:** |  |  |  |  |  | Ketoconazol |
| *Candida albicans* | NA | NA | 13 | NA | 15 | 20 |
| *Aspergillus niger* | NA | NA | NA | NA | 14 | 17 |
