# Supplementary material for: Anti-Alzheimer potential, metabolomic profiling and molecular docking of green synthesized silver nanoparticles of Lampranthus coccineus and Malephora lutea aqueous extracts
Source: PLoS One. 2019 Nov 6;14(11):e0223781. doi: 10.1371/journal.pone.0223781 (PMC6834257; doi:10.1371/journal.pone.0223781)
Supplement: S6 Table — (DOCX) [file pone.0223781.s010.docx]

| **Samples**  **Tested Microorganisms** | ***L. coccineus* aq. extract** | ***L. coccineus* aq. nano extract** | ***M. lutea* aq. extract** | ***M. lutea* aq. nano extract** |
| --- | --- | --- | --- | --- |
| **Gram positive bacteria:** |  |  |  |  |
| *Staphylococcus aureus* | ND | 5000 | 5000 | 1250 |
| *Bacillus sphaericus* | ND | 5000 | ND | 10000 |
| **Gram negative bacteria:** |  |  |  |  |
| *Enterobacter aerogenes* | ND | 5000 | ND | 5000 |
| *Pseudomonas aeruginosa* | ND | 250 | ND | 10000 |
| **Fungi:** |  |  |  |  |
| *Candida albicans* | ND | 250 | ND | 250 |
| *Aspergillus niger* | ND | ND | ND | 5000 |
